# AD/NEURODEGENERATION BLOOD BIOMARKER ASSOCIATIONS WITH PREVALENT AND INCIDENT MOBILITY AND COGNITIVE IMPAIRMENT

**DOI:** 10.1093/geroni/igae098.0005

**Published:** 2024-12-31

**Authors:** B Gwen Windham, Chad Blackshear, Kevin Sullivan, James Pike, Keenan Walker, Thomas Mosley, Priya Palta, Michael Griswold

**Affiliations:** University of Mississippi Medical Center - The MIND Center, Jackson, Mississippi, United States; University of Mississippi Medical Center, Jackson, Mississippi, United States; University of Mississippi Medical Center, Jackson, Mississippi, United States; New York University, New York, New York, United States; National Institute on Aging, Baltimore, Maryland, United States; University of Mississippi Medical Center - The MIND Center, Jackson, Mississippi, United States; University of North Carolina, Chapel Hill, North Carolina, United States; University of Mississippi Medical Center - The MIND Center, Jackson, Mississippi, United States

## Abstract

Blood biomarkers of Alzheimer Disease pathology (Aβ42, Aβ40, p-tau181) and neurodegeneration (neurofilament light (NFL), glial fibrillary acidic protein (GFAP)) may identify individuals at risk for mobility and cognitive impairment. Among 1751 ARIC study participants at Visit 5 (V5, 2011-13, mean age 76.2 years (5.3), 41% men, 28% Black), usual gait speed and cognitive status were assessed over 8 years at V5, V6 (2016-17), and V7 (2017-18). Impairment outcomes were mobility-only (gait speed <0.8m/s, n=430), cognitive-only (dementia, n=38), dual (n=54), or neither (referent, n=1229). Multinomial regression models examined relative risk ratios (RRR) per higher IQR of V5 log-transformed biomarkers with cross-sectional impairments and, among those with neither impairment at V5, incident impairments or death without impairment (n=168) through V7. All models adjusted for V5 age, sex, self-reported race (Black or White), renal function, and body mass index. Aβ42 was reversed and adjusted for Aβ40 (-Aβ42|40) for consistent directions of associations (higher=worse). Higher biomarkers were associated with greater prevalence of mobility, cognitive, and dual impairments. Incident impairments occurred in mobility-only (n=471), cognitive-only (n=39), and dual domains (n=55). All biomarkers were associated with incident dual impairment: p-tau181 (RRR=2.54; 95% CI: 1.45, 4.44), -Aβ42|40 (RRR=1.66; 1.31, 2.09), NfL (RRR=2.69; 1.22, 5.91) and GFAP (RRR=2.13; 1.07, 4.25). P-tau181, -Aβ42|40, and GFAP were also associated with incident cognitive-only impairment; P-tau181, NFL and GFAP were associated with incident mobility-only impairment. AD-pathology versus neurodegeneration blood biomarkers may help identify older adults at differential risk of functional decline, potentially facilitating earlier targeted interventions to preserve mobility and cognitive outcomes.

